# Effect of changing kick frequency on inter-joint coordination during undulatory underwater swimming

**DOI:** 10.1038/s41598-026-57730-9

**Published:** 2026-06-12

**Authors:** Tomohiro Mimura, Yusaku Nakazono, Noriyuki Kida, Hitoshi Koda, Takaaki Tsunokawa

**Affiliations:** 1 Doctoral Programs of Biotechnology, Graduate School of Science and Technology, Kyoto Institute of Technology, Kyoto, Japan; 2 Institute of Health and Sports Science, University of Tsukuba, Ibaraki, Japan; 3 Faculty of Arts and Sciences, Kyoto Institute of Technology, Kyoto, Japan; 4 Advanced Research Initiative for Human High Performance (ARIHHP), University of Tsukuba, Ibaraki, Japan

**Keywords:** Swimming, Undulatory motion, Kick frequency, Coordinative pattern, Vector coding, Physics, Physiology

## Abstract

Undulatory underwater swimming (UUS) contributes to race performance in competitive swimming, yet how inter-joint coordination changes with kick frequency remains unclear. This study examined the effects of kick frequency on trunk–hip and hip–knee inter-joint coordination during UUS using the Modified Vector Coding Technique. Eight male swimmers performed UUS trials at 80–120% of their optimal kick frequency, with kinematics captured using three-dimensional underwater motion analysis. Swimming velocity increased with kick frequency but plateaued beyond 100%. The proportion of the hip–knee anti-phase with distal dominance pattern ––hip and knee rotating in opposite directions with greater angular displacement at the knee–– increased with kick frequency, whereas the absolute durations of this pattern per cycle changed little, indicating that the time required for kick reversal remained nearly constant regardless of kick frequency. Under high-frequency conditions (110% and 120%), preserving the time for kick reversal within a shortened kick cycle was accompanied by constrained trunk undulation during the late downward kick. These findings suggest that when kick frequency is acutely increased beyond the optimal value, hip–knee reversal time is maintained but trunk undulation is impaired, likely contributing to the plateau in swimming velocity.

**Supplementary Information:**

The online version contains supplementary material available at 10.1038/s41598-026-57730-9.

Under World Aquatics regulations, swimmers may swim underwater for up to 15 m after the start and turns in the butterfly, backstroke, and freestyle events. During this phase, almost all swimmers use undulatory underwater swimming (UUS) as their primary means of propulsion, and performance in this segment is a key determinant of race outcome. By reducing wave drag compared with surface swimming, UUS lowers hydrodynamic resistance and enables higher swimming velocity^1^. Mechanically, UUS consists of an undulatory motion that originates in the trunk and propagates to the hip, knee, and ankle joints, with progressively larger distal amplitudes generating propulsion^2,3^. This proximal-to-distal propagation of motion functions as a whip-like kinetic chain, in which energy generated at the trunk is sequentially transmitted through the lower-limb joints to produce high foot velocity^4,5^. Effective transmission of trunk motion to the distal segments through this kinetic chain is therefore essential for efficient propulsion during UUS.

Swimming velocity during UUS generally increases with kick frequency^4^. However, raising kick frequency beyond an individual’s optimal value does not further enhance swimming velocity^6,7^. Under such high kick frequency conditions, Yamakawa et al.^7^ observed increased coactivation of antagonist trunk muscles, suggesting that trunk undulation is constrained. Consistent with this, a simulation study showed that restricting trunk undulation during UUS reduces propulsive efficiency^8^. These findings indicate that excessively high kick frequencies may impair trunk undulation and thereby limit further gains in swimming velocity. However, how trunk and lower-limb undulatory motions change with kick frequency remains unclear.

This chain of undulatory motion is characterized not only by individual joint angles but also by the relative motion between adjacent joints, that is, inter-joint coordination. When trunk undulation is maintained and effectively transmitted to the lower limbs, distal segment velocity increases and propulsion is enhanced^5,8^. Conversely, if the timing of one joint is disrupted or the motion of a particular joint becomes excessively dominant, propagation of the undulatory wave may be interrupted and propulsive efficiency may decrease. Traditional single-joint kinematic variables such as range of motion (ROM) describe how much each joint moves, but not the sequence or dominance with which motion is transmitted from the trunk to the lower limbs. Therefore, to understand how excessively high kick frequencies constrain UUS performance, it is important to clarify how trunk-to-lower-limb undulatory motion is organized (or disrupted) in terms of inter-joint coordination.

In competitive swimming strokes, inter-joint and inter-segmental coordination can account for differences in swimming velocity and stroke strategy^9^, indicating that coordination patterns can characterize performance and technique. In UUS, Tsunokawa et al.^10^ applied the Modified Vector Coding Technique (MVCT) to hip–knee and knee–ankle joint pairs and found that hip–knee anti-phase coordination, in which adjacent joints rotate in opposite directions, was positively correlated with kick frequency. These findings suggest that lower-limb inter-joint coordination contributes to how swimmers achieve higher kick frequencies. However, as described above, UUS performance at high kick frequencies appears to be constrained by impaired trunk undulation rather than by lower-limb coordination alone. Because the undulatory wave in UUS originates in the trunk and propagates distally, trunk–hip coordination may be the first element affected when kick frequency exceeds the optimal value. Despite this, trunk–hip coordination during UUS has not been quantified, nor have frequency-dependent changes in inter-joint coordination been examined. Such quantification is an important step toward understanding how increases in kick frequency alter the propagation of undulatory motion during UUS.

MVCT has been widely applied in motor control research to quantify inter-joint coordination^11,12^. In this technique, an angle–angle diagram is constructed from the time-series data of two adjacent joint angles, and the coupling angle, defined as the orientation of the vector connecting consecutive data points, is calculated at each time point. The coupling angle is then used to classify the relative motion between adjacent joints into four coordinative patterns: in-phase, in which adjacent joints rotate in the same direction, and anti-phase, in which they rotate in opposite directions. Within each category, proximal dominance indicates that the proximal joint exhibits greater angular displacement, whereas distal dominance indicates the reverse. This technique captures the temporal structure of how motion is shared between adjacent joints and is therefore suitable for evaluating the sequential inter-joint coordination during UUS. Clarifying how inter-joint coordination changes with kick frequency is essential for understanding the mechanisms by which excessively high kick frequencies constrain UUS performance. Therefore, this study aimed to clarify the effects of changes in kick frequency on trunk–hip and hip–knee inter-joint coordination during UUS using MVCT. We hypothesised that when kick frequency exceeds the optimal value, trunk ROM decreases and the proportion of trunk-dominant coordinative patterns in trunk–hip coupling decrease, impairing trunk undulation and contributing to the plateau in swimming velocity at excessively high kick frequencies. Coordinative indices obtained may provide coaches with an objective framework for evaluating and optimising kick frequency and undulatory motion in practice.

## Results

### Kinematic variables

Table 1 presents the mean ± SD values of the kinematic variables under each kick frequency condition. Main effects were observed for kick frequency (*F*(4, 28) = 261.92, *p* < .001, *η*² = 0.66), mean swimming velocity (*F*(4, 28) = 3.93, *p* = .011, *η*² = 0.11), and ROM of trunk flexion/extension (*χ²*(4) = 24.7, *p* < .001, *Kendall’s W* = 0.77), hip flexion/extension (*F*(4, 28) = 16.87, *p* < .001, *η*² = 0.71), and knee flexion/extension (*F*(4, 28) = 17.68, *p* < .001, *η*² = 0.72). Post hoc analyses revealed that the mean swimming velocity tended to increase with kick frequency; however, no further increase was observed beyond 100% F. The ROM of the trunk, hip, and knee decreased with increasing kick frequency.

**Table 1:** Kinematic variables at each kick frequency.

Variables	Unit	80% F	90% F	100% F	110% F	120% F	Statistics
Kick frequency	(Hz)	1.71 ± 0.17	1.93 ± 0.21_a_	2.12 ± 0.22_a, b_	2.35 ± 0.25_a, b, c_	2.59 ± 0.30_a, b, c, d_	*p* < .001
Swimming velocity	(m/s)	1.53 ± 0.17	1.51 ± 0.14	1.62 ± 0.13_b_	1.64 ± 0.12_b_	1.60 ± 0.10	*p* = .011
ROM of trunk	(deg)	22.98 ± 3.82	22.06 ± 2.33	19.79 ± 3.14	18.19 ± 3.82	16.68 ± 3.10	*p* < .001
ROM of hip	(deg)	33.83 ± 5.08	32.58 ± 5.02	30.59 ± 6.04	27.31 ± 6.14_a, b_	24.58 ± 4.93_a, b, c_	*p* < .001
ROM of knee	(deg)	76.59 ± 6.99	73.60 ± 8.02	71.47 ± 8.01_a_	67.26 ± 7.56_a, b_	61.41 ± 10.39_a, b, c, d_	*p* < .001
ROM of ankle	(deg)	32.90 ± 7.85	33.79 ± 7.66	36.51 ± 10.64	30.78 ± 5.65	29.65 ± 7.71	n.s.

Values are mean ± SD. Superscript letters indicate significant pairwise differences after Bonferroni correction (*p* < .05): a, difference from 80%F; b, difference from 90%F; c, difference from 100%F; d, difference from 110%F. n.s., no significant main effect (*p* ≥ .05). Friedman test was used for trunk ROM; one-way repeated-measures ANOVA was used for all other variables. ROM, range of motion. F, kick frequency condition expressed as a percentage of each swimmer’s optimal kick frequency.

### Inter-joint coordination

**Fig. 1:**
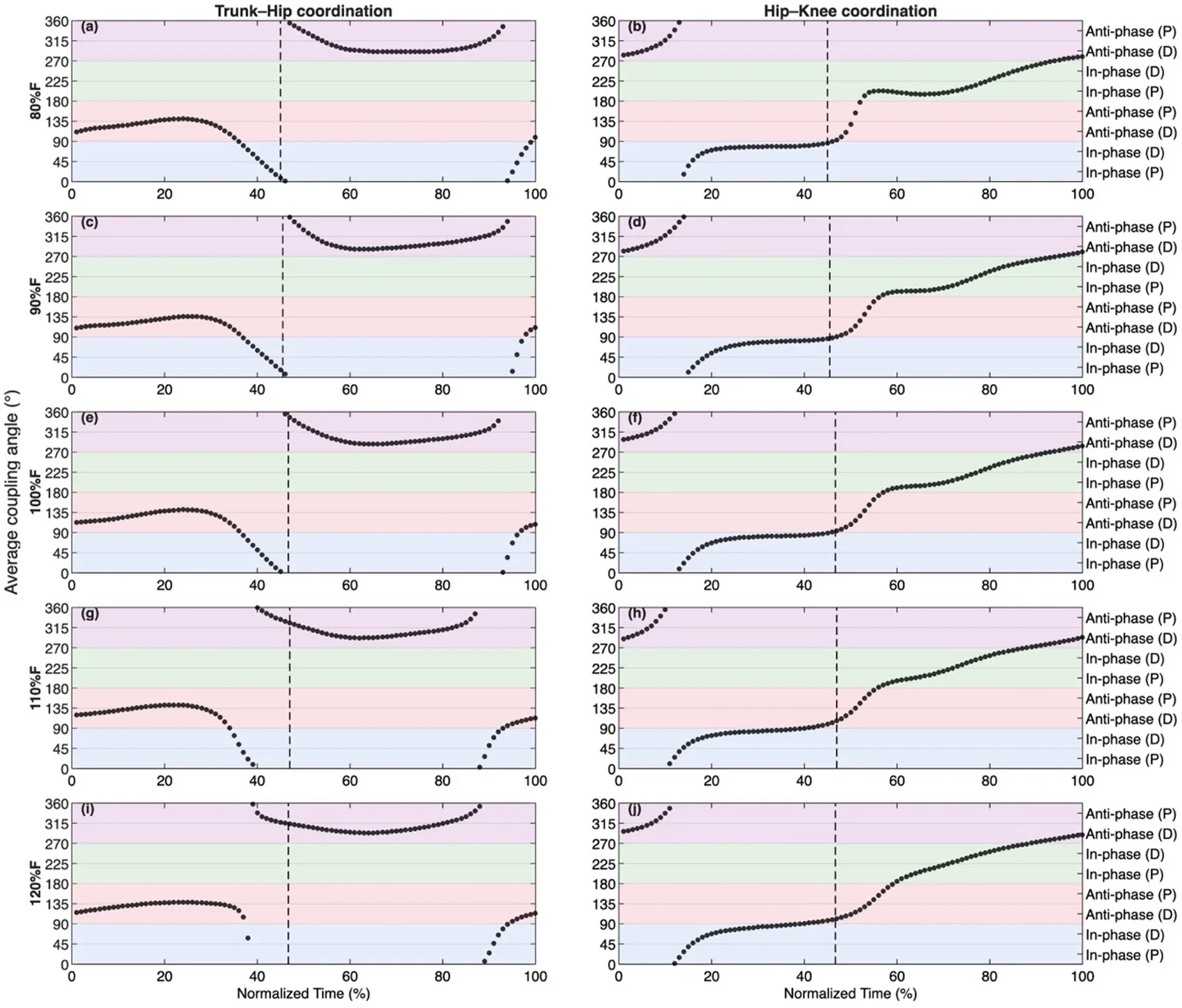
Average coupling angle during one kick cycle at each kick frequency condition for the trunk–hip and hip–knee joint pairs. Left column (**a**, **c**, **e**, **g**, **i**): trunk–hip coordination at 80% F, 90% F, 100% F, 110% F, and 120% F, respectively. Right column (**b**, **d**, **f**, **h**, **j**): hip–knee coordination at 80% F, 90% F, 100% F, 110% F, and 120% F, respectively. Each dot represents the average coupling angle at a normalised time point, calculated across eight participants using circular statistics. Background shading indicates the coordination pattern zones: blue (0–90°), in-phase; red (90–180°), anti-phase; green (180–270°), in-phase; purple (270–360°), anti-phase. The dashed vertical line indicates the average transition time from the downward kick to the upward kick for each condition.

Figure 1 illustrates the time-series changes in the inter-joint coordination across kick frequency conditions. In the trunk–hip joint pair, a reduction in the occurrence of the in-phase coordinative pattern was observed at approximately 40% of the normalized cycle time (late downward kick phase) at 110% F and 120% F. The occurrence rates were 9% at 80–100% F, 5% at 110% F, and 1% at 120% F. In the hip–knee joint pair, increasing kick frequency led to greater occurrence of anti-phase (D) patterns within the D50-P50 to D100-P0 range during both the early and late portions of the downward kick and upward kick phases.

Table 2 presents the median [IQR] values of the inter-joint coordinative patterns for each kick frequency condition. Main effects were observed in the hip–knee joint pair for the proportions of in-phase (P) and anti-phase (D) patterns (*F* (4, 28) = 9.35, *p* < .001, *η*² = 0.57; *F* (4, 28) = 24.33, *p* < .001, *η*² = 0.78). The proportion of in-phase (P) was higher at 80% F than at 110% F (*p* = .034) and 120% F (*p* = .027), and the distribution of the medians showed an overall decreasing trend with increasing kick frequency. In contrast, the proportion of anti-phase (D) was higher at 110% F and 120% F than at 80% F and 90% F (*p* < .001), and 110% F was higher than that at 100% F (*p* = .046). Table 3 presents the absolute durations of each coordinative pattern per kick cycle across the five kick frequency conditions. In the trunk–hip joint pair, the absolute durations of in-phase patterns decreased with increasing kick frequency. In the hip–knee joint pair, the absolute durations of anti-phase (D) showed only small between-condition differences and did not show a systematic increase with kick frequency, despite the marked increase in its proportion across conditions (Table 2).

**Table 2:** Proportion of coordinative patterns at each kick frequency.

Variables	Unit	80%F	90%F	100%F	110%F	120%F	Statistics
*Trunk - hip*							
In-phase (P)	(%)	7.0 [2.2]	5.0 [0.8]	4.0 [3.0]	3.0 [2.8]	3.5 [1.0]	n.s.
In-phase (D)	(%)	8.5 [3.0]	9.0 [3.0]	6.0 [2.5]	6.5 [2.8]	5.0 [2.5]	n.s.
Anti-phase (P)	(%)	16.5 [10.5]	20.0 [14.8]	21.5 [23.0]	12.5 [14.2]	25.0 [17.0]	n.s.
Anti-phase (D)	(%)	69.5 [13.2]	67.0 [13.5]	67.5 [24.5]	79.5 [20.2]	66.0 [14.2]	n.s.
*Hip - knee*							
In-phase (P)	(%)	20.0 [12.5]	18.5 [7.2]	17.0 [9.0]	17.0 [5.2] _a_	13.0 [4.5] _a_	*p* < .001
In-phase (D)	(%)	51.5 [11.5]	51.0 [5.0]	47.0 [4.5]	44.0 [8.0]	44.5 [7.0]	n.s.
Anti-phase (P)	(%)	6.0 [1.5]	8.5 [4.2]	9.5 [1.2]	9.0 [1.0]	9.5 [4.2]	n.s.
Anti-phase (D)	(%)	20.0 [3.8]	22.0 [3.5]	26.5 [6.5]	30.5 [3.8] _a, b, c_	34.0 [4.0] _a, b_	*p* < .001

Values are median [IQR]. Superscript letters indicate significant pairwise differences after Bonferroni correction (*p* < .05): a, difference from 80%F; b, difference from 90%F; c, difference from 100%F; d, difference from 110%F. n.s., no significant main effect (*p* ≥ .05). One-way repeated-measures ANOVA was used for variables satisfying normality after centred log-ratio transformation; Friedman test was used for all others. In-phase indicates that adjacent joints rotate in the same direction; anti-phase indicates rotation in opposite directions. (P) and (D) denote proximal and distal dominance, respectively, referring to which joint exhibits greater angular displacement.

**Table 3:** Absolute durations of trunk–hip and hip–knee coordinative patterns per kick cycle at each kick frequency.

Variables	Unit	80%F	90%F	100%F	110%F	120%F	Statistics
*Trunk-hip*							
In-phase (P)	(s)	0.04 [0.02]	0.03 [0.02]	0.02 [0.02]	0.02 [0.01]	0.01 [0.02]	*p* < .001
In-phase (D)	(s)	0.05 [0.02]	0.05 [0.02]	0.03 [0.03]	0.03 [0.03]	0.02 [0.02]	*p* < .001
Anti-phase (P)	(s)	0.11 [0.10]	0.10 [0.10]	0.11 [0.12]	0.05 [0.09]	0.11 [0.08]	n.s.
Anti-phase (D)	(s)	0.41 [0.16]	0.36 [0.14]	0.32 [0.12]	0.30 [0.12]	0.27 [0.11]	*p* = .005
*Hip - knee*							
In-phase (P)	(s)	0.12 [0.11]	0.11 [0.07]	0.09 [0.06]	0.08 [0.03]	0.05 [0.03]	*p* < .001
In-phase (D)	(s)	0.30 [0.04]	0.27 [0.05]	0.23 [0.04]	0.19 [0.05]	0.17 [0.06]	*p* < .001
Anti-phase (P)	(s)	0.04 [0.02]	0.05 [0.02]	0.04 [0.02]	0.04 [0.02]	0.04 [0.02]	n.s.
Anti-phase (D)	(s)	0.12 [0.03]	0.11 [0.03]	0.12 [0.04]	0.13 [0.03]	0.13 [0.02]	n.s.

Values are median [IQR]. Friedman test was used for all variables. n.s., no significant main effect (*p* ≥ .05). No pairwise comparisons reached significance after Bonferroni correction. In-phase indicates that adjacent joints rotate in the same direction; anti-phase indicates rotation in opposite directions. (P) and (D) denote proximal and distal dominance, respectively, referring to which joint exhibits greater angular displacement.

## Discussion

As shown in Table 1, the participants successfully synchronized their kick cycles with the prescribed frequencies using a metronome. The mean swimming velocity increased with kick frequency but showed no further gains beyond 100% F. In addition, the ROM of the trunk, hip, and knee decreased with increasing kick frequency. These results are consistent with previous reports, confirming that the observed changes represent characteristic kinematic adjustments during higher kick frequency UUS^6,7,13^.

With increasing kick frequency, the proportion of in-phase (P) patterns in the hip–knee joint pair decreased, whereas the proportion of anti-phase (D) patterns increased. Anti-phase (D) was concentrated at the beginning and end of both the downward and upward kicks (Fig. 1), whereas its absolute duration did not change substantially across conditions (Table 3). Tsunokawa et al.^10^ reported that, during kick reversal, the change in rotational direction of the hip joint precedes that of the knee joint, and that anti-phase coordination in this phase is strongly associated with kick frequency. Anti-phase (D) in the present study was similarly concentrated during the kick reversal phases, suggesting that proximal joints play a role in preparing the reversal of motion in distal joints within the whip-like kinetic chain of UUS. The finding that the absolute duration of anti-phase (D) remained nearly constant regardless of kick frequency suggests that the preparatory motion for reversing the rotational direction between proximal and distal joints requires a minimum time that cannot be further shortened. Therefore, when swimmers acutely increase their kick frequency, the strategy employed is to shorten the overall kick cycle while maintaining the time allocated to kick reversal, and consequently the time available for phases other than kick reversal is compressed.

However, because no further increases in mean swimming velocity were observed under high-frequency conditions, the influence of other factors, particularly trunk–hip coordination, must be considered. At 100% F, a characteristic sequence of coordinative patterns was observed across the kick cycle (Fig. 1): anti-phase in the early downward kick, in-phase in the late downward kick, anti-phase in the early upward kick, and a shift from in-phase to anti-phase in the late upward kick. When kick frequency was increased to 110% F, the occurrence of trunk-extension/hip-flexion in-phase coordination in the late downward kick was markedly reduced, and this reduction became even more pronounced at 120% F. The temporal trade-off described above, in which kick reversal time was maintained while the overall cycle duration decreased, resulted in the greatest compression occurring in the late downward kick. This compression constrained the relative motion between the trunk and hip in this phase, impairing trunk undulatory motion. Nakashima^8^ demonstrated through simulation that restricting trunk undulation during UUS leads to reduced propulsive efficiency and swimming velocity, and the changes in trunk–hip coordination observed in the present study are consistent with this finding. Accordingly, the constraint on trunk undulation arising from this temporal trade-off may be one of the factors limiting further increases in swimming velocity under high kick frequency conditions. However, the relationship between trunk undulation and swimming velocity likely involves multiple mediating factors, including the influence on foot velocity through the whip-like kinetic chain and changes in propulsive force, and the present kinematic data alone cannot establish this causal pathway.

Previous studies have reported that the constraint of trunk undulation accompanying increases in kick frequency manifests as muscular coactivation in the trunk^7^; however, how the temporal distribution of motion between the trunk and lower limbs changes with kick frequency had not been clarified. In the present study, the application of MVCT to sagittal-plane flexion/extension movements enabled us to capture changes in trunk–hip coordination accompanying acute increases in kick frequency as a trade-off in the temporal distribution of motion between joint pairs. This finding would be difficult to obtain from conventional single-joint kinematics alone and demonstrates the utility of MVCT analysis in quantifying the temporal structure of relative motion between adjacent joints.

The present study analysed changes in inter-joint coordination from a kinematic perspective. However, UUS performance is also influenced by hydrodynamic factors. For example, the Strouhal number is a dimensionless parameter relating kick frequency, kick amplitude, and swimming velocity, while Froude efficiency is an index of propulsive efficiency calculated from the ratio of swimming velocity to body wave velocity^4,6^. In addition, the flow characteristics around the swimmer depend on the Reynolds number and drag forces. Shimojo et al.^6^ reported that Froude efficiency decreased with increasing kick frequency, and Yamakawa et al.^7^ reported both a decrease in Froude efficiency and an increase in trunk muscle coactivation. The changes in trunk–hip coordination observed under high kick frequency conditions in the present study may be related to such decreases in propulsive efficiency and musculoskeletal constraints. However, the analysis of hydrodynamic parameters and muscle activity was beyond the scope of the present study, which focused specifically on the temporal structure of inter-joint coordination. From a practical standpoint, the coordinative pattern indices obtained in this study may serve as an objective framework for evaluating swimmers’ kick technique. For example, swimmers who show a marked reduction in trunk–hip in-phase patterns when kick frequency is increased may be experiencing constrained trunk undulation, and training that focuses on improving trunk–lower-limb coordination rather than simply increasing kick frequency may be beneficial. Future studies integrating MVCT-based coordination analysis with hydrodynamic indices and electromyographic measurements could provide a more comprehensive understanding of the relationships among movement coordination, propulsive efficiency, and muscle activity during UUS. In addition, future studies could complement underwater motion capture with wearable inertial measurement units, which have been used to assess kinematic changes in competitive swimming^14^, to examine the stability of coordinative patterns across multiple consecutive kick cycles.

## Limitations

This study has several limitations that should be acknowledged. First, the sample size was small (*n* = 8). Although this is comparable to previous studies on UUS^6,7^ and a post hoc power analysis indicated adequate statistical power for the main outcomes, the small sample limits the generalisability of the findings. Second, the analysis was based on a single kick cycle per condition per swimmer. While this approach is consistent with the constraints of the filming area used for three-dimensional motion capture, it does not account for potential cycle-to-cycle variability in coordinative patterns. Third, the swimmers examined in this study had a relatively high competitive level, with an average World Aquatics Point score of 757. Therefore, the present findings are primarily limited to elite swimmers, and caution is required when generalising the results to swimmers with different performance levels. Fourth, in this study, MVCT was applied to sagittal plane flexion/extension movements to evaluate the coordinative structure of wave propagation from the trunk to the lower limbs, which constitutes the primary propulsive motion during UUS. However, joint rotations in the transverse and frontal planes also occur during UUS, and recent studies have reported that hip internal/external rotation contributes to propulsion^13,15^. Because MVCT can be applied to rotations of adjacent joints within the same movement plane, incorporating inter-joint coordination analyses in the transverse and frontal planes in future studies would contribute to a more comprehensive understanding of the movement mechanisms underlying UUS.

## Conclusion

This study aimed to clarify the effects of changes in kick frequency on inter-joint coordination during UUS using MVCT. With increasing kick frequency, the proportion of hip–knee anti-phase (D) patterns increased, whereas the absolute durations of this pattern within a kick cycle changed little. This suggests that the time required for kick reversal was largely maintained across conditions. Under high-frequency conditions, however, this maintenance of kick reversal time was accompanied by a marked reduction in trunk–hip in-phase patterns in the late downward kick. This indicates that trunk undulatory motion was constrained, which may have been one of the factors limiting furture increases in swimming velocity. By quantifying the temporal structure of inter-joint coordination, MVCT enabled the detection of changes in the distribution of motion between joint pairs accompanying acute increases in kick frequency. These findings provide insight into why further increases in kick frequency do not necessarily lead to higher swimming velocity.

## Materials and methods

### Participants

Eight male competitive swimmers participated in this study (age: 21.3 ± 2.4 years, height: 1.73 ± 0.03 m, body mass: 73.7 ± 5.1 kg, World Aquatics points: 757.8 ± 42.7). The participants specialized in butterfly, freestyle, or backstroke; breaststroke specialists were excluded because breaststroke does not involve consecutive UUS during the underwater phase. The World Aquatics points were calculated based on each participant’s personal best record at the time of the experiment in their specialized event. All participants practiced UUS as part of their daily training. The sample size used in this study was comparable to that of previous studies on UUS^6,7^. In addition, a post hoc power analysis conducted using G*Power 3.1 indicated that the statistical power (1-β) for the main outcomes of this study ranged approximately from 0.62 to 1.00. Prior to the experiment, the purpose, procedures, and potential risks were fully explained, both in writing and orally, and written informed consent was obtained from all participants. This study was approved by the Research Ethics Committee of the University of Tsukuba (No. 023–131) and was conducted in accordance with the Declaration of Helsinki.

### Experimental setting and data acquisition

**Fig. 2:**
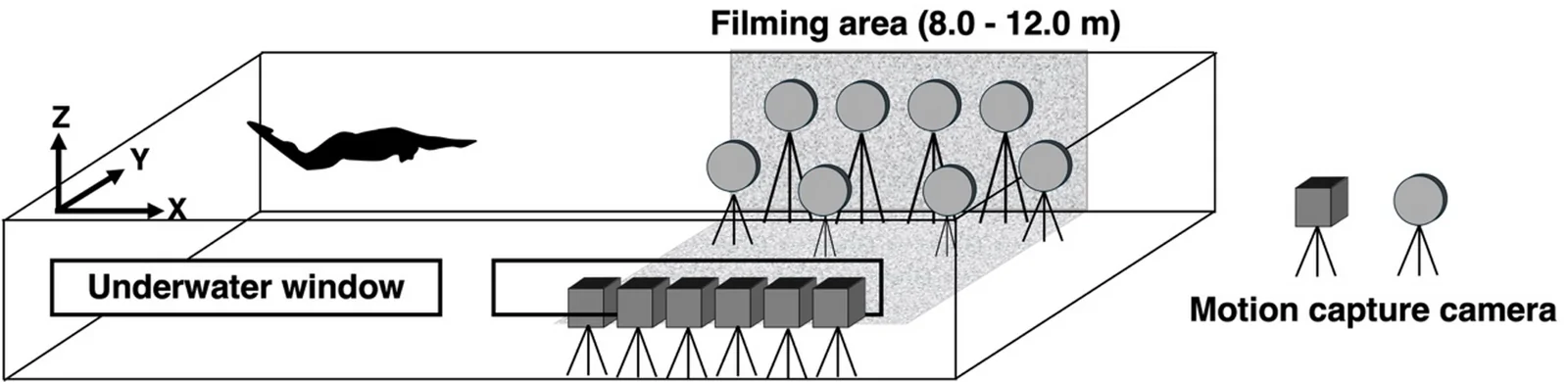
Experimental setting. Fourteen underwater cameras of a three-dimensional motion analysis system were positioned to capture the swimming motion within the filming area. The global coordinate system was defined with the X-axis in the swimming direction, the Y-axis in the transverse direction, and the Z-axis in the vertical direction.

**Fig. 3:**
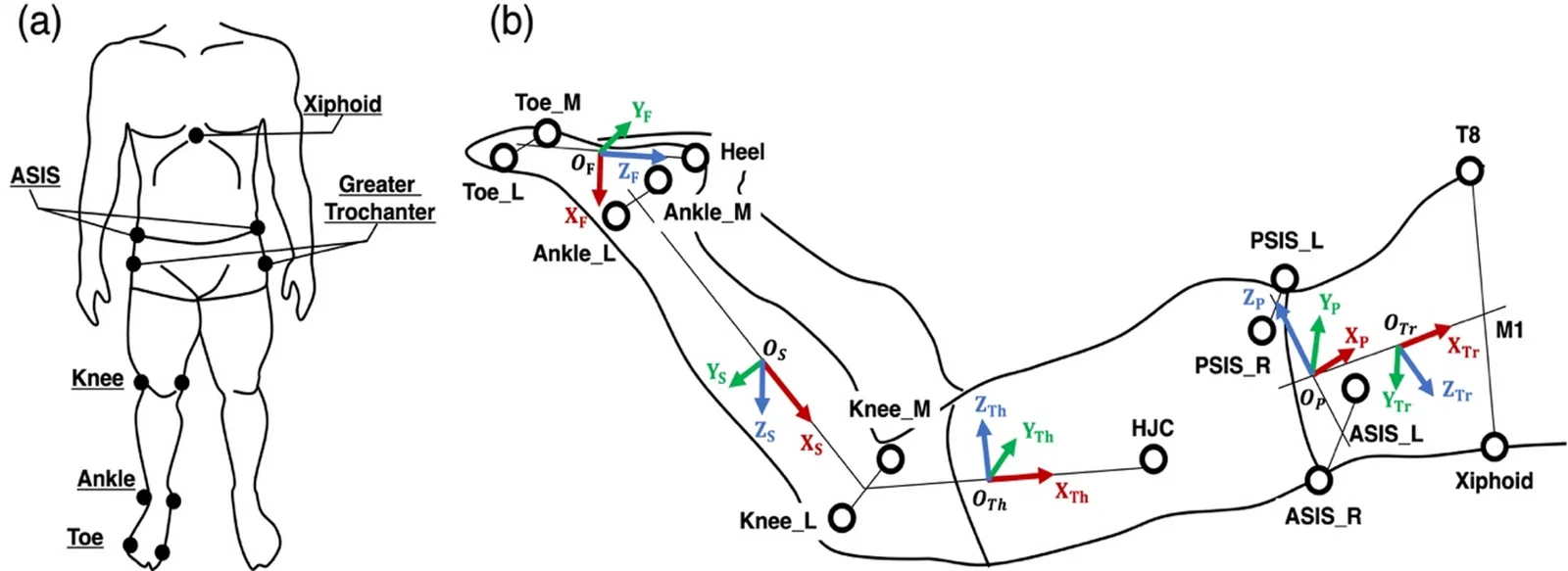
(**a**) Approximate positions of the anatomical landmarks on the body surface. T8, PSIS, and Heel are located on the posterior surface of the body. (**b**) Definition of the segment coordinate systems. Open circles indicate the anatomical landmarks on which LED markers were placed. Dashed circles indicate computed points. Local coordinate systems were defined for five body segments: trunk (Tr), pelvis (P), right thigh (Th), right shank (S), and right foot (F). The origin (O), X-axis (red), Y-axis (green), and Z-axis (blue) are shown for each segment. Abbreviations: T8, 8th thoracic vertebra; M1, midpoint between T8 and xiphoid process (the most caudal point on the sternum); HJC, hip joint centre; ASIS, anterior superior iliac spine; PSIS, posterior superior iliac spine; Toe_M and Toe_L, 1st and 5th metatarsal heads; Ankle_M and Ankle_L, medial and lateral malleoli; Knee_M and Knee_L, medial and lateral femoral epicondyles.

The experiment was conducted in an indoor pool (50 m × 7 lanes, depth: 1.35–3.80 m, water temperature: 27.4 ± 0.5 °C). All trials were performed in the shallow section, where the water depth was 1.35 m. Body landmark coordinates during swimming were captured with a three-dimensional (3D) motion analysis system (VENUS 3D, Nobby Tech Inc., Japan) and reconstructed with its dedicated software. Fourteen cameras surrounding the swimmer recorded at 100 Hz (Fig. 2). Calibration accuracy was assessed from the residuals between reconstructed and true inter-marker distances on the calibration object, and the standard error was < 0.4 mm. A right-handed global coordinate system was defined with the water surface as the origin, swimming direction as the X-axis, transverse direction as the Y-axis, and vertical direction as the Z-axis. Fifteen LED markers (Kirameki, Nobby Tech Inc., Japan) were attached to anatomical landmarks defined with reference to the ISB recommendations^16,17^ (Fig. 3a, b). 3D marker coordinates were filtered using a low-pass Butterworth filter with a cut-off frequency of 6 Hz, which was determined by residual analysis over a wide range of cut-off frequencies^18^.

### Swim trials

After completing a 30 min self-selected warm-up, each swimmer performed six prone UUS trials over a distance of 15 m, starting with an underwater push-off from the wall. During the trials, swimmers were instructed to maintain a depth greater than 0.5 m below the water surface to minimize the effects of wave drag^19^. In the first trial, the swimmers were instructed to cover a distance of 15 m as quickly as possible. From the 3D coordinate data obtained in this trial, the duration of one kick cycle was determined using VENUS 3D as the time from one peak to the next of the Z coordinates at the midpoint between first and fifth metatarsal heads.

Kick frequency was then calculated as the reciprocal of the cycle duration. Five kick frequency conditions were established based on these values: 80%, 90%, 100%, 110%, and 120% of an individual’s kick frequency (80%, 90%, 100%, 110%, and 120% F). From the second trial onward, the swimmers performed trials under the five kick frequency conditions. Before and during each trial, metronome beeps corresponding to the target kick frequency were provided through a waterproof metronome device (Tempo Trainer Pro, FINIS Inc., USA) attached to the cap of the swimmer. Swimmers were instructed to synchronize their kick cycle with the metronome beeps and complete the 15 m distance as quickly as possible. The trials under the five kick frequency conditions were performed in a randomized order, with at least 3 min of rest between trials. Before each trial, swimmers were given sufficient practice time to ensure synchronization of their kick cycles with the metronome beeps. A trial was considered valid if the kick frequency during swimming was within ± 3% of the target.

### Calculation of kinematic parameters

The kinematic variables for a one-kick cycle were calculated from the 3D coordinates obtained using the motion capture system. In this study, a kick cycle was divided into two phases: the downward kick phase, defined as the interval from the maximum to the minimum value of the toe Z-coordinate, and the upward kick phase, defined as the interval from the minimum to the subsequent maximum value of the toe Z-coordinate. Mean swimming velocity was defined as the average velocity in the X-axis direction of the midpoint between the greater trochanters.

To quantify the joint angles during swimming, local coordinate systems were defined for five body segments: trunk, pelvis, right thigh, right shank, and right foot (Supplementary Table S1; Fig. 3b). Because UUS is a bilaterally symmetrical movement in which both legs move simultaneously, only the right side was analysed. The centre of the hip joint was estimated from the coordinates of the anterior superior iliac spines and the greater trochanter of the femur in accordance with the recommendations of the Clinical Gait Analysis Forum of Japan^20^. Based on these local coordinate systems, rotation matrices between adjacent segments were calculated for each frame. These rotation matrices were decomposed into Cardan angles in the XYZ sequence to obtain the joint angles. During UUS, vertical toe velocity is a key determinant of propulsive force and is produced through sagittal-plane joint rotations propagating distally via a whip-like kinetic chain^4,5^. Because the primary undulatory motion is thus generated by flexion/extension about the mediolateral axis, this study focused on sagittal plane flexion/extension movements, and only the rotation angle around the Y-axis was extracted and used for analysis, defined as trunk flexion (−)/extension (+), hip flexion (+)/extension (−), knee flexion (−)/extension (+), and ankle plantarflexion (−)/dorsiflexion (+). Each joint angle was time normalized to 100% of one kick cycle using cubic spline interpolation. The ROM of each joint was calculated as the difference between the maximum and minimum values at each joint angle.

### Quantification of inter-joint coordination by MVCT

**Fig. 4:**
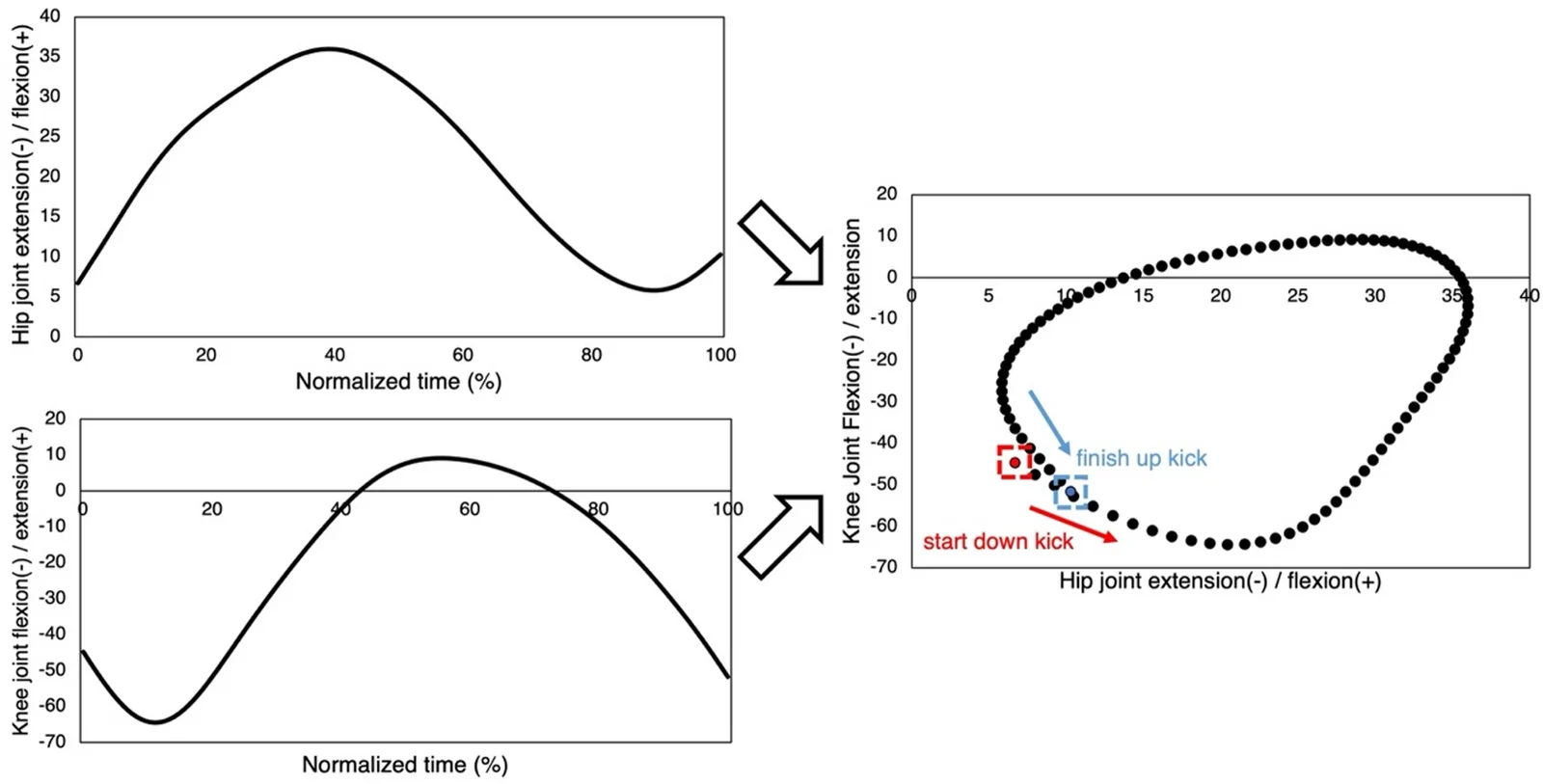
The process from joint angle time series to an angle–angle diagram. The upper left panel shows the hip joint flexion (+) /extension (−) angle and the lower left panel shows the knee joint flexion (−) / extension (+) angle over a normalised kick cycle for a representative participant at 100% F. The right panel shows the angle–angle diagram constructed by plotting the hip joint angle on the horizontal axis and the knee joint angle on the vertical axis. Each dot represents one normalised time point. The red circle with a dashed border indicates the start of the downward kick and the blue circle with a dashed border indicates the end of the upward kick.

To quantify the inter-joint coordination during UUS, we applied MVCT to the trunk–hip and hip–knee joint pairs, adapting the procedure of Needham et al.^11^ for this task. Although 3D kinematic data were collected, inter-joint coordination was quantified in the sagittal plane. MVCT defines in-phase and anti-phase relationships on the basis of angle–angle plots of joint rotations within the same movement plane, and the present study focused on sagittal plane flexion/extension as the primary propulsive motion during UUS. First, angle–angle diagrams were constructed by plotting the proximal joint angles (${\theta }_{P\left(i\right)}$, ${\theta }_{P(i+1)}$) on the x-axis and the distal joint angles (${\theta }_{D\left(i\right)}$, ${\theta }_{D(i+1)}$) on the y-axis. The coupling angle (${\gamma }_{i}$), defined as the orientation of the vector formed by two consecutive data points in the time series (*i*), was then calculated (Eqs. 1–3). As shown in Fig. 4, the transformation from an angle–angle trajectory to a coupling angle can be visualized step-by-step, clarifying how the relative joint motion is represented over time.

1 $${\gamma }_{i} = A\mathrm{tan}\left(\frac{{\theta }_{D(i+1)}-{\theta }_{D\left(i\right)}}{{\theta }_{P(i+1)}-{\theta }_{P\left(i\right)}}\right)\cdot \frac{180}{\pi } {\theta }_{P(i+1)}-{\theta }_{P\left(i\right)} >0$$

2 $${\gamma }_{i} = A\mathrm{tan}\left(\frac{{\theta }_{D(i+1)}-{\theta }_{D\left(i\right)}}{{\theta }_{P(i+1)}-{\theta }_{P\left(i\right)}}\right)\cdot \frac{180}{\pi }+180 {\theta }_{P(i+1)}-{\theta }_{P\left(i\right)}<0$$

3 $${\gamma }_{i}=\left\{\begin{array}{c}{\gamma }_{i}=90 {\theta }_{P(i+1)}-{\theta }_{P\left(i\right)}=0\,and\,{\theta }_{D\left(i+1\right)}-{\theta }_{D\left(i\right)}>0\\ \begin{array}{c}{\gamma }_{i}=-90 {\theta }_{P\left(i+1\right)}-{\theta }_{P\left(i\right)}=0\,and \,{\theta }_{D\left(i+1\right)}-{\theta }_{D\left(i\right)}<0\\ {\gamma }_{i}=-180 {\theta }_{P\left(i+1\right)}-{\theta }_{P\left(i\right)}<0\,and\,{\theta }_{D\left(i+1\right)}-{\theta }_{D\left(i\right)}=0\end{array}\\ {\gamma }_{i}=Undefined {\theta }_{P(i+1)}-{\theta }_{P\left(i\right)}=0\,and\,{\theta }_{D\left(i+1\right)}-{\theta }_{D\left(i\right)}=0\end{array}\right.$$

Next, the average coupling angle ($\bar{\gamma} _{i}$) was calculated using circular statistics based on the mean horizontal (${\bar{x}} _{i}$) and vertical (${\bar{y}} _{i}$) components at each time point (Eqs. 4 and 5)^21,22^.

4 $$\overline{{x_{i} }} = \frac{1}{n}\mathop \sum \limits_{{i = 1}}^{n} \cos \gamma _{i}$$

5 $$\overline{{y_{i} }} = \frac{1}{n}\mathop \sum \limits_{{i = 1}}^{n} \sin \gamma _{i}$$

Finally, the average coupling angle ($\bar{\gamma }_{i}$) was adjusted by applying Eq. (6), which constrained the values within a range of 0° to 360°. The average coupling angle was used to compare the time-series profiles of inter-joint coordination across kick frequency conditions.

6 $$\overline{{\gamma _{i} }} = \left\{ {\begin{array}{*{20}l} {A\tan \left( {\frac{{\overline{{y_{i} }} }}{{\overline{{x_{i} }} }}} \right) \cdot \frac{{180}}{\pi }~\,x_{i} > 0,~y_{i} > 0} \hfill \\ {A\tan \left( {\frac{{\overline{{y_{i} }} }}{{\overline{{x_{i} }} }}} \right) \cdot \frac{{180}}{\pi } + 180~\,x_{i} < 0} \hfill \\ {A\tan \left( {\frac{{\overline{{y_{i} }} }}{{\overline{{x_{i} }} }}} \right) \cdot \frac{{180}}{\pi } + 360\,x_{i} > 0,~y_{i} < 0} \hfill \\ {90\,x_{i} = 0,~y_{i} > 0} \hfill \\ { - 90\,x_{i} = 0,~y_{i} < 0} \hfill \\ {undefined~~~~~x_{i} = 0,~y_{i} = 0} \hfill \\ \end{array} } \right.$$

**Fig. 5:**
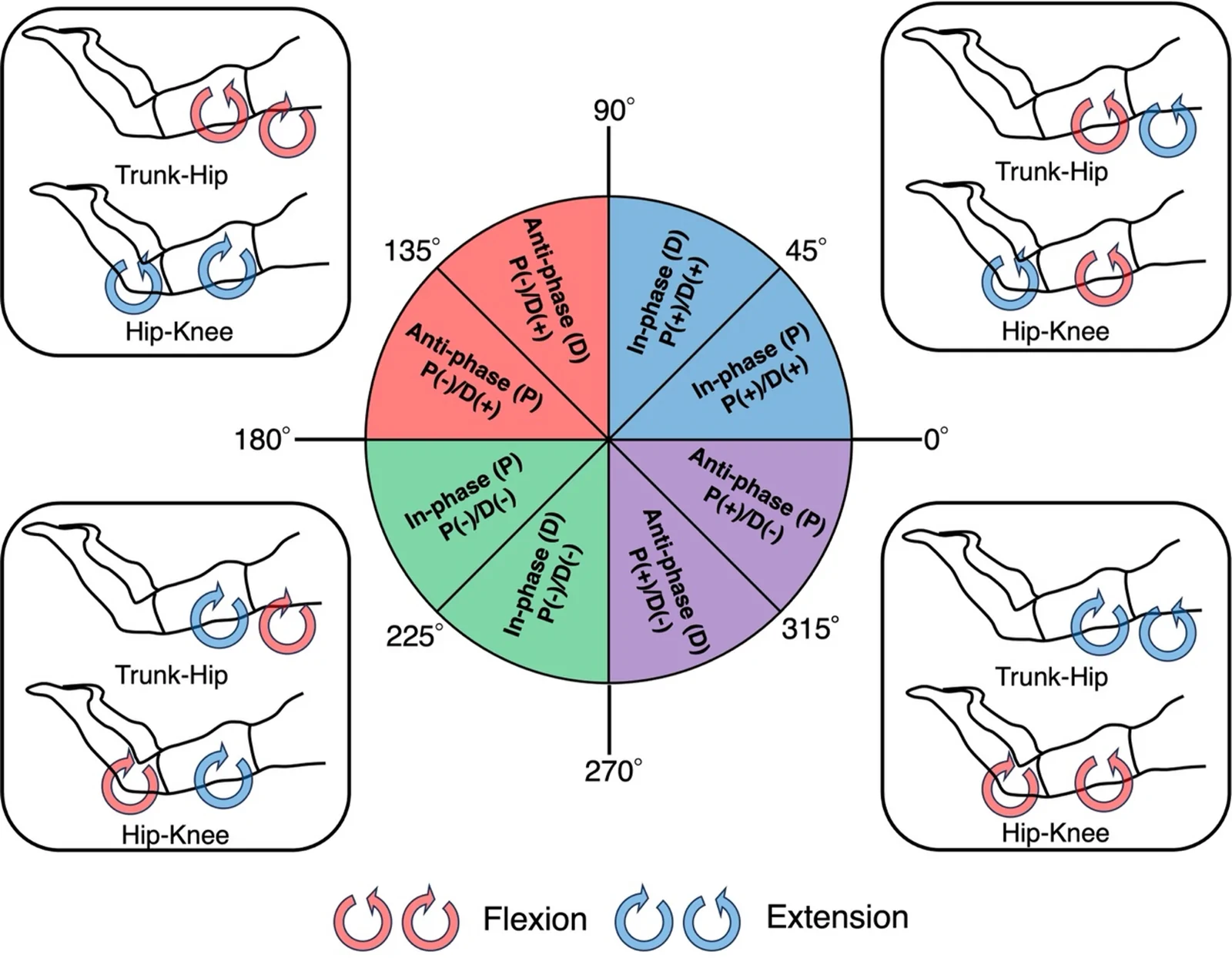
Classification of coordinative patterns based on the coupling angle. The circular diagram shows the eight coordination categories defined by the Modified Vector Coding Technique. In-phase coordination (blue and green regions) indicates that adjacent joints rotate in the same direction, whereas anti-phase coordination (red and purple regions) indicates rotation in opposite directions. Within each category, proximal (P) and distal (D) dominance are distinguished by the relative magnitude of angular displacement. The illustrations at the four corners depict representative trunk–hip and hip–knee joint configurations for each quadrant. Red arrows indicate flexion and blue arrows indicate extension.

Following Needham et al.^12^, the coupling angle at each normalised time point for each participant was classified into four coordinative patterns: in-phase with proximal dominance, in-phase with distal dominance, anti-phase with proximal dominance, and anti-phase with distal dominance (Fig. 5). Here, proximal and distal dominance are abbreviated as P and D, respectively. For brevity, these four patterns are denoted as in-phase (P), in-phase (D), anti-phase (P), and anti-phase (D). When the adjacent joints rotated in the same direction during joint actions that moved the toe vertically downward or upward, the motion was defined as in-phase, whereas the opposite directions of rotation were defined as anti-phase. Within each of these categories, proximal dominance was defined when the proximal joint angle exhibited greater displacement than the distal joint angle, whereas distal dominance was defined when the distal joint angle exhibited greater displacement than the proximal joint angle. Based on this classification, the occurrence proportion of each coordinative pattern was calculated for a kick cycle. In addition, the absolute durations in each coordinative pattern within a kick cycle were obtained by multiplying its proportion by the kick-cycle duration.

### Statistical analysis

Kinematic variables were expressed as mean ± SD for each kick frequency condition. The proportions and absolute durations of coordinative patterns were expressed as median [IQR]. Considering the compositional nature of the coordinative pattern data, a centred log-ratio (CLR) transformation was applied to the proportions only. The normality of kinematic variables, CLR-transformed components, and absolute durations of coordinative patterns were assessed using the Shapiro-Wilk test. For variables with normal distributions, a one-way repeated-measures analysis of variance (ANOVA) was conducted. The assumption of sphericity was tested using Mauchly’s test, and when violated, the Greenhouse-Geisser correction was applied. Post-hoc comparisons were performed using Bonferroni-adjusted multiple comparisons. For variables that did not satisfy normality, the Friedman test was used, followed by Wilcoxon signed-rank tests with Bonferroni correction for post-hoc analysis. For variables showing differences, effect sizes (η² or Kendall’s W) were also reported to indicate the magnitude of effects. All statistical analyses were performed using R software (ver. 4.4.3), with the alpha level set at 5%. All tests were two-tailed.

## Supplementary Information

Below is the link to the electronic supplementary material.

Supplementary Material 1

## Data Availability

The datasets generated and analysed during the current study are available from the corresponding author on reasonable request.
