# Supplementary material for: Effect of changing kick frequency on inter-joint coordination during undulatory underwater swimming
Source: Sci Rep. 2026 Jun 12;16:26770. doi: 10.1038/s41598-026-57730-9 (PMC13507235; doi:10.1038/s41598-026-57730-9)
Supplement: Supplementary file 1 [file 41598_2026_57730_MOESM1_ESM.pdf]

Definitions of local coordinate systems.

Segment	Origin	Base vector V	X-axis	Y-axis	Z-axis
Pelvis	Midpoint between anterior superior iliac spines & posterior superior iliac spines centers (O_P)	O_P to midpoint of left anterior superior iliac spines-posterior superior iliac spine	Cross product of V & Z	Cross product of X & Z	O_P to midpoint of posterior superior iliac spines
Trunk	Midpoint between M1 (8th thoracic vertebra-xiphoid process) & O_p (O_{Tr})	8th thoracic vertebra to xiphoid process	M1 to O_{Tr}	Cross product of X & V	Cross product of X & Y
Right thigh	Midpoint between center of the hip joint & knee center (O_{Th})	lateral femoral epicondyle to medial femoral epicondyle	O_{Th} to center of the hip joint	Cross product of X & Z	Cross product of X & V
Right shank	Midpoint between knee & ankle centers (O_S)	Medial malleolus to lateral malleolus	O_S to knee center	Cross product of X & Z	Cross product of X & V
Right foot	Midpoint between toe center & calcaneus (O_F)	O_F to ankle center	Cross product of Y & Z	Cross product of V & Z	O_F to calcaneus
